
JOURNAL INFORMATION
==============================
NLM Title Abbreviation: Exploration (Beijing)
Journal ID: Exploration (Beijing)
NLM Journal ID: 9918383883006676
Journal ID: EXP2

Exploration

ISSN: 2766-8509
EISSN: 2766-2098
Publisher: Wiley

ARTICLE INFORMATION
==============================
PMCID: PMC13094516
DOI: 10.1002/exp2.70158
Article ID: EXP270158
Article version: 1
Subjects: Issue Information, Issue Information

Issue Information

EXPLORATION EDITORS

Electronic publication date: 2026 Apr 20
Publication date: 2026 Apr
Volume: 6
Issue: 2
Electronic Location ID: 270158
Copyright: © 2026 The Author(s). Exploration published by Henan University and John Wiley & Sons Australia, Ltd.
License: This is an open access article under the terms of the http://creativecommons.org/licenses/by/4.0/ License, which permits use, distribution and reproduction in any medium, provided the original work is properly cited.
License URL: https://creativecommons.org/licenses/by/4.0/

Figure count: 0
Table count: 0
Page count: 8

source-schema-version-number: 2.0
cover-date: April 2026
details-of-publishers-convertor: Converter:WILEY_ML3GV2_TO_JATSPMC version:6.7.3 mode:remove_FC converted:20.04.2026

==============================

### Senior Editorial Board Members

**William A. Banks**, *University of Washington, Seattle, USA*
**Cyrille Boyer**, *University of New South Wales, Sydney, Australia*
**Ashley Bush**, *The University of Melbourne, Melbourne, Australia*
**Yihai Cao**, *Karolinska Institutet, Stockholm, Sweden*
**Chunying Chen**, *National Center for Nanoscience and Technology, Beijing, China*
**Guoping Chen**, *National Institute for Materials Science, Tsukuba, Japan*
**Xuesi Chen**, *Changchun Institute of Applied Chemistry, CAS, Changchun, China*
**Thomas Davis**, *The University of Queensland, Queensland, Australia*
**Kenneth Dawson**, *University College Dublin, Dublin, Ireland*
**Chunhai Fan**, *Shanghai Jiao Tong University, Shanghai, China*
**Omid C. Farokhzad**, *Harvard Medical School, Boston, USA*
**David Galbraith**, *University of Arizona, Tucson, USA*
**Ewa Goldys**, *University of New South Wales, Sydney, Australia*
**J. Justin Gooding**, *University of New South Wales, Sydney, Australia*
**Ning Gu**, *Southeast University, Nanjing, China*
**Zaiping Guo**, *The University of Adelaide, Adelaide, Australia*
**Zijian Guo**, *Nanjing University, Nanjing, China*
**Taeghwan Hyeon**, *Seoul National University, Seoul, Korea*
**Mietek Jaroniec**, *Kent State University, Kent, USA*
**Lei Jiang**, *Beihang University, Beijing, China*
**Il-Doo Kim**, *Korea Advanced Institute of Science and Technology, Republic of Korea*
**Dan Li**, *The University of Melbourne, Melbourne, Australia*
**Yuehe Lin**, *Washington State University, USA*
**Wolfgang Parak**, *Universität Hamburg, Hamburg, Germany*
**Shi-Zhang Qiao**, *The University of Adelaide, Adelaide, Australia*
**Alan Rowan**, *The University of Queensland, Brisbane, Australia*
**Chun-Peng Song**, *Henan University, Kaifeng, China*
**Martina Stenzel**, *University of New South Wales, Sydney, Australia*
**Ben Zhong Tang**, *The Hong Kong University of Science and Technology, Hong Kong, China*
**Pall Thordarson**, *University of New South Wales, Sydney, Australia*
**Joseph Wang**, *University California, San Diego, USA*
**Xuelu Wang**, *Henan University, Kaifeng, China*
**Paul Weiss**, *University of California, Los Angeles, USA*
**Andrew Whittaker**, *The University of Queensland, Brisbane, Australia*
**Xiyun Yan**, *Institute of Biophysics, CAS, Beijing, China*
**Yadong Yin**, *University of California, San Francisco, USA*
**Juyoung Yoon**, *Ewha Womans University Seoul Korea*
**Shu-Hong Yu**, *University of Science and Technology of China, Hefei, China*
**Kourosh Kalantar Zadeh**, *The University of Sydney, Sydney, Australia*
**Gaiping Zhang**, *Henan Agricultural University, Zhengzhou, China*
**Dongyuan Zhao**, *Fudan University, Shanghai, China*
**Yuliang Zhao**, *National Center for Nanoscience and Technology, Beijing, China*

### Editorial Board Members

**Massimo Bottini**, *University of Rome Tor Vergata, Rome, Italy*
**Weibo Cai**, *University of Wisconsin – Madison, Madison, USA*
**Jun Chen**, *University of Wisconsin – Madison, Madison, USA*
**Hangrong Chen**, *Shanghai Institute of Ceramics, CAS Shanghai, China*
**Zhe-Sheng (Jason) Chen**, *St. John's University, New York, USA*
**João Diogo Osório de Castro Conde**, *NOVA Medical School, Universidade Nova de Lisboa, ToxOmics, United Kingdom*
**Daxiang Cui**, *Shanghai Jiao Tong University, Shanghai, China*
**Zongqiang Cui**, *Wuhan Institute of Virology, CAS, Wuhan, China*
**Zhifei Dai**, *Peking University, Beijing, China*
**Yizhou Dong**, *Icahn School of Medicine at Mount Sinai, Mount Sinai, USA*
**Rawil Fakhrullin**, *Kazan Federal University, Kazan, Russia*
**Wei Gao**, *California Institute of Technology, USA*
**Zhen Gu**, *Zhejiang University, Hangzhou, China*
**Peixuan Guo**, *The Ohio State University, Ohio, USA*
**Gang Han**, *University of Massachusetts Medical School, Worcester, USA*
**Xiaojing Hao**, *University of New South Wales, Sydney, Australia*
**Yu Han**, *King Abdullah University of Science and Technology (KAUST), Saudi Arabia*
**Tony Hu**, *Tulane University, New Orleans, USA*
**Xingyu Jiang**, *Southern University of Science and Technology, Shenzhen, China*
**Yan Jiao**, *University of Adelaide, Australia*
**Yan Jin**, *Air Force Medical University, China*
**Deling Kong**, *Nankai University, Tianjin, China*
**Jun-Seok Lee**, *Korea University, Seoul, Korea*
**Ki-Bum Lee**, *The State University of New Jersey, New Jersey, USA*
**David Tai Wei Leong**, *National University of Singapore, Singapore*

### Exploration

Open Access

| | |
|---|---|
| **Editor-in-Chief** | Xing-Jie Liang, *National Center for Nanoscience and Technology, Beijing, China* |
| **Executive Editor-in-Chief** | Bingyang Shi, *Joint Centre for Biomedical Innovation, Macquarie University, Sydney, Australia* |
| **Deputy Editors** | Yu Chen, *Shanghai University, Shanghai, China*<br>Kelong Fan, *Institute of Biophysics, CAS, Beijing, China*<br>Huiyu Liu, *Beijing University of Chemical Technology, Beijing, China*<br>Wei Tao*, Harvard Medical School, Boston, USA*<br>Qingyu Yan, *Nanyang Technological University, Singapore, Singapore* |
| **Associate Editors** | Meiwan Chen, *University of Macau, Macau, China*<br>Jianxun Ding, *Changchun Institute of Applied Chemistry, CAS, China*<br>Zhi-Gang Chen, *Queensland University of Technology, Queensland, Australia*<br>Jing Jing Duan, *Nanjing University of Science and Technology, Nanjing, China*<br>Zi (Sophia) Gu, *University of New South Wales, Sydney, Australia*<br>Yuanyu Huang, *Beijing Institute of Technology, Beijing, China*<br>Ho Won Jang, *Seoul National University, Seoul, Korea*<br>Xiaoyuan Ji, *Tianjin University, Tianjin, China*<br>Linlin Li, *Beijing Institute of Nanoenergy and Nanosystems, Chinese Academy of Sciences, Beijing, China*<br>Han Lin, *Shanghai Institute of Ceramics Chinese Academy of Sciences, Shanghai, China*<br>Daishun Ling, *Shanghai Jiao Tong University, Shanghai, China*<br>Chengliang Wang, *Huazhong University of Science and Technology, Wuhan, China*<br>Jiajia Xue, *Beijing University of Chemical Technology, Beijing, China* |
| **Editorial Office** | editorial@exploration-journals.com |

**AIMS AND SCOPE**

*Exploration* is an international, cross-disciplinary and high-quality open-access journal, aiming to promote the development of interdisciplinary sciences and technologies by publishing state-of-the-art research articles, communications, reviews, perspectives and commentaries.

*Exploration* focuses on crossing the boundaries of conventional research disciplines, disseminating impactful research to the general public, and inspiring global multidisciplinary collaborations. *Exploration* envisions to provide insights for future science and technology, to catalyze novel integrations of cross-disciplinary studies, as well as to explore new insights and methods to overcome the obstacles for a sustainable future for society.

*Exploration* welcomes the comprehensive articles on cutting-edge studies utilizing the perspectives and tools from all research fields. Topics of particular interest include, but are not limited to the following:

* biology and biotechnology
* medicine and biomedical engineering
* chemistry
* material sciences
* physics and quantum technology
* nanoscience and technology
* optics, photonics, electronics and robotics
* energy science and technology
* engineering
* mathematics and computer sciences
* emerging interdisciplinary research

**Disclaimer:** The Publisher, Henan University, and Editors cannot be held responsible for any errors in or any consequences arising from this journal. The views and opinions expressed do not necessarily reflect those of the Publisher, Henan University, and the Editors, neither does the publication of advertisements constitute any endorsement by the Publisher, Henan University, Editors, or Authors of the products advertised.

**Guanna Li**, *Wageningen University, Wageningen, Netherlands*
**Qin Li**, *Griffith University, Brisbane, Australia*
**Yaping Li**, *Shanghai Institute of Materia Medica, CAS, Shanghai, China*
**Kang Liang**, *The University of New South Wales (UNSW), Sydney, Australia*
**Gang Liu**, *Xiamen University, Xiamen, China*
**Juewen Liu**, *Waterloo University, Waterloo, Canada*
**Zhuang Liu**, *Soochow University, Suzhou, China*
**Jonathan Lovell**, *University at Buffalo, State University of New York, New York, USA*
**Olivia Merkel**, *Ludwig-Maximilians-Universität München, München, Germany*
**Michael J. Mitchell**, *University of Pennsylvania, Philadelphia, USA*
**Guangjun Nie**, *National Center for Nanoscience and Technology, Beijing, China*
**Amirali Popat**, *The University of Queensland, Brisbane, Australia*
**Ling Peng**, *Aix Marseille University, CNRS, Center Interdisciplinaire de Nanoscience de Marseille, Marseille, France*
**Kanyi Pu**, *Nanyang Technological University, Nanyang, Singapore*
**Zhiyong Qian**, *Sichuan University, Chengdu, China*
**Xiaogang Qu**, *Changchun Institute of Applied Chemistry, CAS, Changchun, China*
**Shisong Rong**, *Harvard Medical School, Cambridge, USA*
**Zongping Shao**, *Nanjing Tech University, Nanjing, China*
**Youqing Shen**, *Zhejiang University, Hangzhou, China*
**Jing Sun**, *Griffith University, Gold Coast, Australia*
**Liangliang Sun**, *Michigan State University, East Lansing, USA*
**Xun Sun**, *Sichuan University, Chengdu, China*
**Hang Ta**, *Griffith University, Brisbane, Australia*
**Peter Timashev**, *Sechenov University, Moscow, Russia*
**Krasimir Vasilev**, *Flinders University, Adelaide, Australia*
**Ernst Wagner**, *Ludwig-Maximilians-Universität München, München, Germany*
**Changyong Wang**, *Academy of Military Medical Sciences, Beijing, China*
**Hongxia Wang**, *Queensland University of Technology, Brisbane, Australia*
**Jun Wang**, *South China University of Technology, Guangzhou, China*
**Qiangbin Wang**, *Suzhou Institute of Nano-Tech and Nano-Bionics, CAS, Suzhou, China*
**Gareth R. Williams**, *UCL School of Pharmacy, London, UK*
**Aiguo Wu**, *Ningbo Institute of Materials Technology and Engineering, CAS, Ningbo, China*
**Jingwei Xie**, *University of Nebraska Medical Center, Omaha, USA*
**Benggang Xing**, *The Hong Kong Polytechnic University, Hong Kong, China*
**Gordon Xu**, *The University of Queensland, Brisbane, Australia*
**Qiaobing Xu**, *Tufts University, Medford, USA*
**Xue Xue**, *Nankai University, China*
**Zhimou Yang**, *Nankai University, Tianjin, China*
**Yusuke Yamauchi**, *Nagoya University, Japan*
**Cunjiang Yu**, *Pennsylvania State University, Philadelphia, USA*
**Quan Yuan**, *Wuhan University, Wuhan, China*
**Yanglun Yu**, *Chinese Academy of Forestry, China*
**Shanqing Zhang**, *Griffith University, Gold Coast, Australia*
**Zhihong Zhang**, *Huazhong University of Science and Technology, Wuhan, China*
**Chun-Xia Zhao**, *The University of Adelaide, Adelaide, Australia*
**Yanli Zhao**, *Nanyang Technological University, Nanyang, Singapore*
**Guizhi (Julian) Zhu**, *University of Michigan, Ann Arbor, USA*
**Rongrong Zhu**, *School of Life Sciences and Technology, Tongji University, Shanghai, China*

**Copyright:** All articles published by *Exploration* are fully open access: immediately freely available to read, download and share. All articles are published under the terms of the Creative Commons Attribution License (CC-BY). The CC-BY license permits use, distribution and reproduction in any medium, provided the original work is properly cited and allows the commercial use of published articles. Copyright on articles published under a Creative Commons license in a Wiley Open Access journal is retained by the author(s). Authors grant Wiley a license to publish the article and identify itself as the original publisher. Authors also grant any third party the right to use the article freely within the terms of the specified license as long as its integrity is maintained and its original authors, citation details and publisher are identified. The Wiley Open Access Agreement formalizes these and other terms and conditions of publishing articles. Full details can be found at www.wileyopenaccess.com.

**For submission instructions, subscription, and all the latest information, visit** http://www.wileyonlinelibrary.com/journal/exploration.

Exploration
Open Access

### Contents

**Volume 6, Issue 2** **April 2026**

### Commentary

**When Rare Is Not Small: Amyotrophic Lateral Sclerosis Initiatives and Therapy** .......... 70114
*Yang Liu, Xue Xia, Yingfang Chen, Haoying Yang, Xiaochen Chen, Lei Cai, and Bingyang Shi*

**Dendritic Cell-T Cell Crosstalk Mediated Cancer Immunotherapy** .......... 70134
*Ying Cai, Jiameng Chen, and Yaping Li*

### Review

**Microengineering the Liver: Strategies for Constructing Functional Liver-on-a-Chip Devices** .......... 70137
*Jie Wang, Ziwei Liang, Jiapu Wang, Zongyi Li, Shaojie Wang, Yan Wei, Xin Xie, and Di Huang*

**The Metabolic Role of Mitochondria in the Perinatal Cardiac Development and Cardiovascular Diseases** .......... 20240414
*Minghao Li, Ruiyi Zhang, Xinyuan Li, and Hefeng Huang*

**Micro Pattern-Based 3D Cell Culture Platform: An Overview of Technologies and Applications** .......... 20240469
*Xinglong Zhu, Yi Li, Hulin Long, Yijia Wu, Ningwei Sun, Shun Li, Yiyao Liu, Huiqi Xie, and Ji Bao*

**Recent Advances in Conjugated Microporous Polymers for Photocatalysis: Mechanism, Synthesis and Applications** .......... 20240487
*Dun Zhou, Kongqing Zhang, Xiaobai Li, Yongpeng Liu, Liang Yao, and Hongwei Ma*

### Research Article

**Bacterial Biohybrids With Dual Magnet and Hypoxia Tropism for Ferroptosis Activation in Deep Tumor Regions** .......... 20240287
*Sijie Shao, Huilan Zhuang, Tingjie Bai, Xuemei Zeng, Angelo Homayoun All, and Shuangqian Yan*

**Collaborative Optimization of Electromagnetic Interference Shielding, Adaptive Multi-Color, and Thermal Camouflage of Basalt Fibers by Temperature-Induced Gradient Structure Control** .......... 70135
*Sijie Qiao, Zhicheng Shi, Annan He, Zhiyu Huang, Aixin Tong, Binhao Wang, Jun He, Jiaxin Wang, Wei Ke, Na Yao, Shichao Zhao, Yong Qin, Weilin Xu, and Fengxiang Chen*

**Autologous Extracellular Matrix-Based Cell-Free Therapy for Tissue Regeneration Through Trem2$^{+}$ Macrophages Mediated Angiogenesis** .......... 20250031
*Mengmeng Hou, Nini Shi, Yajie Guo, Jiezhang Tang, Han Peng, Baoyan Liang, Yixuan Yu, Chenggang Yi, and Huichen Li*

**Immunosuppressive Microenvironment Reprogramming by Synergistic Sonodynamic Therapy of Phthalocyanine-MOF Hybrids for Hepatocellular Carcinoma** .......... 20250074
*Han Wu, Lihui Gu, Jiahao Xu, Chuyue Zhang, Mingda Wang, Chao Li, Lanqing Yao, Yongkang Diao, Yuchen Li, Fujie Chen, Huixuan Fan, Yuze Zhao, Feng Shen, and Tian Yang*

**PSMA-Targeting Macrophage Membrane-Coated Nanoparticles for Precision Diagnosis and Combination Therapy of Prostate Cancer** .......... 20240393
*Keying Zhang, Bo Gao, Jingwei Wang, Yu Li, Chao Xu, Fa Yang, Shaojie Liu, Hongji Li, Chao Zhang, Xiangliang Meng, Zhaokun Shi, Rui Zhang, Ruili Zhang, Zhongliang Wang, Weihong Wen, Qiang Zhang, Yuankang Zou, Donghui Han, Huijie Bian, and Weijun Qin*

**Ultrasmall $Au_{10-12}$ Nanoclusters: A Novel Platform for $^{223}Ra$-Targeted Tumor $\alpha$-Therapy** .......... 70144
*Yifei Jiang, Huizhen Yang, Guoping Jia, Wangxi Hai, Rongyi Huang, Peng Wang, Min Zhang, Danni Li, Yizhou Chen, Xiao Li, Biao Li, and Chunfu Zhang*

**Rumen Mycobiome Thiamine Metabolism Contributes to Subacute Rumen Acidosis Tolerance in Goats Through Enhancing Epithelial Cell Proliferation via IGFBP2/IGF1 Axis Activation** ................ 70142
*Jingyi Xu, Xiaodong Chen, Yi Ma, Jianrong Ren, Guanghao Xu, Peilin Zhang, Wenhao Jia, Quanqiu Deng, Yuquan Xiao, Junhu Yao, and Shengru Wu*

**EnzRetro: Enzymatic Retrosynthetic Planning With Site-Specific Reaction Edits Based on Sequence Generative Architecture** ................ 70129
*Yahui Cao, Haoshu Chen, Tao Zhang, Xin Zhao, Bingzhi Li, and Xiangrui Zheng*

**Modification-Response Integrated Modules Driven Cyclization-Dependent Prodrug Self-Assembly for Reshaping Pro-Apoptotic Tumor Redox Homeostasis** ................ 20240448
*Yixin Sun, Shiyi Zuo, Wenfeng Zang, Lingxiao Li, Xianbao Shi, Mingzhong Li, Zhonggui He, Bingjun Sun, and Jin Sun*

**Visible Photosensitizing Disinfectant Spray for Combating Multidrug-Resistant *Candidozyma Auris* in Healthcare Settings** ................ 20250276
*Xiaoyu Xu, Ming-Yu Wu, Baoping Li, Jie Li, Siyu Chen, Luojia Chen, Donghu Yu, Liupiaopiao Yang, Ziyu Hong, Huaqin Pan, Wei Xiang, Shun Feng, Jong Seung Kim, Lianrong Wang, Zhiqiang Li, Shi Chen, and Meijia Gu*

**Surmounting Recalcitrant Airway Inflammatory Disorders With a Multi-Targeting Therapeutic Strategy by Tannic Acid-Modified $CuInP_2S_6$ Nanosheets** ................ 20250073
*Ming Liu, Changyi Xu, Renqiang Ma, Xinran Xie, Ying Xie, Mengya Du, Zhaofeng Xu, Shuaiyin Zhang, Jian Li, Weiping Wen, and Zhaoxu Tu*

**Spherical Nucleic Acid Stabilized Cage Type Three-Dimensional Electrochemiluminescence Probe for Sensitive and Amplification-Free Diagnosis of Infectious Diseases** ................ 20240451
*Yu Fu, Wenlu Song, Jinyan Lin, Wei Wang, Lunjing Liu, Wenjie Wu, Huiyi Yang, Jingru Wang, Yang Yang, Bowen Shu, Yideng Jiang, and Yuhui Liao*

**Flexible Multi-Mode Electrochromic Displays for Human Motion Monitoring and Pattern Display in Dark Environments** ................ 20240444
*Xue Chen, Qi Zhao, Li Wang, Xin Yu, Xu Liang, Zhang Chen, John Wang, and Yanfeng Gao*

***RBFOX1* Dysfunction Unlocks *APOE4*-Associated Microglial Genesis and Exacerbates Alzheimer's Pathology in Human Cerebral Organoids** ................ 70160
*Bowen Zhang, Changjie Shi, Jiayi Zhao, Qiuhong Hua, Houchun Zhang, Hailin Gao, Yuanyuan Qian, Jiaxue Cha, Jing Li, Jiayao Chen, Tae-Hyung Kim, Jianhuang Xue, Yujun Hou, and Ru Zhang*

**Real-Time Feedback Strategically Regulates Optoelectronics for Customized Optogenetic Spinal Cord Regeneration** ................ 20250138
*Kaishun Xia, Runze Hu, Xiaopeng Zhou, Jiangjie Chen, Xinmao You, Jingkai Wang, Hao Li, Yiqing Tao, Chao Yu, Haibin Xu, Yuang Zhang, Kesi Shi, Yi Li, Chenggui Wang, Fangcai Li, Chengzhen Liang, Ying Chen, and Qixin Chen*

### Editor-in-Chief

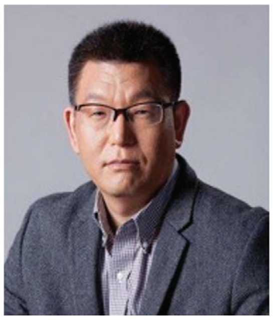

**Xing-Jie Liang**, *National Center for Nanoscience and Technology, Beijing, China*

Dr. Xing-Jie Liang got Ph.D. at National Key Laboratory of Biomacromolecules, Institute of Biophysics, Chinese Academy of Sciences. He finished his postdoc with Dr. Michael M. Gottesman, member of NAS and Deputy Director of NIH, for 5 years at Laboratory of Cell Biology, Center for Cancer Research, National Cancer Institute, National Institutes of Health. Then he worked as a Research Fellow at Surgical Neurology Branch, NINDS, NIH. Dr. Liang is currently a principal investigator at Center for Excellence in Nanoscience, Chinese Academy of Sciences. As current president of Chinese Association of Nanobiology, he is the elected Fellow of American Institute for Medical and Biological Engineering (AIMBE) and Faculty Member of F1000 Research (Pharmacology & Drug Delivery). Dr. Liang has successfully developed “Injectable Nanomicelle Powder with Irinotecan” approved with CFDA and transferred NDA to pharmaceutical industry for clinical trials. Dr. Liang’s research interests are in elucidating mechanisms to improve drugability and nanomedicinal bioavailability by nanotechnology in vitro and in vivo, and novel strategies to increase therapeutic efficiency on cancers and infective diseases. Developing drug delivery strategies for prevention/circumvent of clinical adaptive treatment tolerance (ATT) is current program ongoing in Dr. Liang’s lab based on the understanding of basic physio-chemical and biological processes of nanomedicine.

### Executive Editor-in-Chief

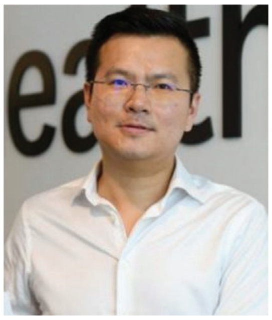

**Bingyang Shi**, *Joint Centre for Biomedical Innovation, Macquarie University, Sydney, Australia*

After receiving his Ph.D. in the University of Adelaide in 2014, Dr. Shi was awarded Macquarie University Research Fellowship in 2015 and joined Department of Biomedical Sciences to build an independent Brain Drug Delivery Research Group with the supervision of senior mentors. With supporting with numbers of competitive grants (e.g., NHMRC-ARC Dementia Research Development Fellowship, Australian Endeavour Leadership Fellowship, Mason Foundation project, NHMRC project grants and Investigator grant), his Brain Drug Delivery Research Group has been growing to be a Joint Centre for Biomedical Innovation.

Dr. Shi’s research ambition is to address a major challenge in the treatment of brain diseases – which is the successful delivery of therapeutic agents across the Blood Brain Barrier (BBB) – the BBB acts as a cellular barrier that prevents toxins, foreign materials and other dangerous molecules from entering the brain, however as a consequence this poses major challenges for getting therapeutic molecules into the brain. To overcome this, he has developed several core patented technologies for brain delivery systems (e.g., Bio-NPs and Glu-NPs), set up collaboration networks across multiple countries, and made close connections with industry and hospital partners, towards practical solutions.

### Deputy Editors

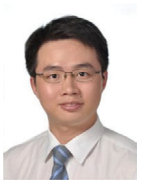

**Yu Chen**, *Shanghai University, Shanghai, China*

Prof. Yu Chen received his Ph.D. degree at Shanghai Institute of Ceramics, Chinese Academy of Sciences (SICCAS). He was the full professor in SICCAS. He is now the full professor in Shanghai University. His research focuses on materdicine, nanomedicine and nanobiotechnology, which involve the design, fabrication, biomedical application and clinical translation of versatile biomaterials, including drug/gene delivery, molecular imaging, chemoreactive nanomedicine, energetic nanomedicine, theragenerative biomaterials, nanodynamic therapy and localized disease therapy. He is 2018, 2019 and 2020 Highly Cited Researcher by Clarivate Analytics, Web of Science.

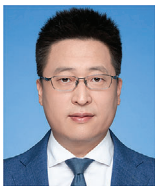

**Kelong Fan**, *Institute of Biophysics, CAS, Beijing, China*

Prof. Kelong Fan received his Ph.D. degree in cell biology from the Institute of Biophysics, Chinese Academy of Sciences in 2014. After which he stayed to further pursue 3 years postdoc training and 2 years of associate professor work experience before attaining a full professor position in 2019. He is interested in exploring the novel functions and applications of nanozyme in biomedicine, with top priority to design functional nanozyme by learning from nature, and to develop novel strategies for disease theranostics. He now serves as Associate Editor of *Exploration* and Guest Editor of *Frontiers in Chemistry*.

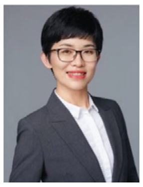

**Huiyu Liu**, *Beijing University of Chemical Technology, Beijing, China*

Huiyu Liu completed her Ph.D. degree in the Technical Institute of Physics and Chemistry (TIPC) under the Chinese Academy of Sciences (CAS). Afterward, she worked as an associate professor at TIPC before becoming a full professor and PI of Bionanomaterials & Translational Engineering Laboratory at Beijing University of Chemical Technology in late 2015. Dr. Liu's research primarily concentrates on the synthesis, characterization, and analysis of various materials' fundamental properties and their application in the medical field. She has authored and co-authored over 110 peer-reviewed journal articles, and Google Scholar gives a citation of 9,900 times with an h-index of 48. Currently, she serves as the Deputy Editor of *Exploration* and an Editorial Board Member of *Mater. Des.*, *Mater. Express*, and *BMEMat*. Dr. Liu's website: www.liuhuiyugroup.com.

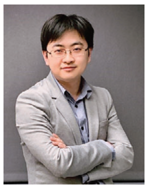

**Wei Tao,** *Harvard Medical School, Boston, USA*

Prof. Wei Tao is the Farokhzad Family Distinguished Chair for Innovation (Endowed Chair), and Principal Investigator in the Center for Nanomedicine at Brigham and Women's Hospital, Harvard Medical School. He is also the first Chaired Professor as an assistant professor in his institution. He has published over 150 papers in prestigious journals including *Nature*/*Science*/*Cell Press*/*PNAS* family journals, and he is a Clarivate's Global Highly Cited Researcher (continuously selected since 2021). Prof. Tao's research interests include biomaterials, nanotechnology and drug delivery, as well as their various applications in translational medicine.
Prof. Tao has received multiple awards/grants including the American Lung Association Lung Cancer Discovery Award (2023), American Heart Association (AHA) Transformational Project Award (2023), AHA Collaborative Sciences Award (2019), Harvard/Brigham Anaesthesia Department Basic Scientist Grant (2018), Nanotechnology Foundation (2022), Khoury Innovation Award (2019), Center for Nanomedicine Research Fund (2019), Stepping Strong Breakthrough Innovator Award (2020), U.S. METAvivor Early Career Investigator Award (2018), MIT Technology Review Top Chinese Innovators Under 35 (2020), *Chemical Society Reviews* Emerging Investigator Award (2022), *Advanced Materials* Rising Star (2022), *Materials Today* Rising Star Award (2021), *Angewandte Chemie* Most Outstanding Referees Award (2021, 2022), *Materials Horizons* Top 10 Outstanding Reviewers Award (2019), World's Top 2% Scientist (continuously selected since 2019), *etc.* He also serves on numerous editorial boards including as Founding Editor-in-Chief of *Biomedical Technology* (Elsevier, KeAi's new journal); Deputy Editor of *Exploration* (Wiley), Associate Editor of *Journal of Nanobiotechnology* (Springer Nature & BMC) and *Aggregate* (Wiley); Guest Editor of *eLife*, *etc.*; and Editorial/Advisory Board Member of *Matter* (Cell Press), *etc.*

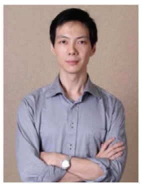

**Qingyu Yan**, *Nanyang Technological University, Singapore, Singapore*

Qingyu Yan is currently a professor in School of Materials Science and Engineering in Nanyang Technological University. He obtained his B.Sc. in Materials Science and Engineering, Nanjing University. He finished his Ph.D. from Materials Science and Engineering Department of State University of New York at Stony Brook. After that, He joined the Materials Science and Engineering Department of Rensselaer Polytechnic Institute as a postdoctoral research associate. He joined School of Materials Science and Engineering of Nanyang Technological University as an assistant professor in early 2008 and became a Professor in 2018.

### Associate Editors

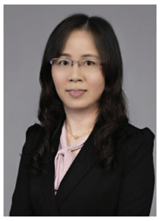

**Meiwan Chen**, *University of Macau, Macau, China*

Prof. Meiwan Chen received her Ph.D. degree in Pharmaceutics from Sun Yat-sen University in 2010. After training as a postdoc for 2 years, she joined Institute of Chinese Medical Science as an assistant professor at the University of Macau in 2012, and was further promoted to associate professor and full professor in 2018 and 2022, respectively. Her research focuses on biomaterial and nanomedicine, especially on development of novel drug delivery systems for active ingredients from Chinese medicine. She now serves as an Associate Editor of *Exploration*.

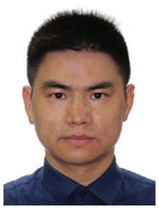

**Zhi-Gang Chen**, *Queensland University of Technology, Queensland, Australia*

Zhi-Gang Chen is a founding director for the ARC Research Hub in Zero-emission Power Generation for Carbon Neutrality and a Capacity Building Professor in Energy materials, and Academic Research Lead in the School of Chemistry and Physics at the Queensland University of Technology. He got his Ph.D. in materials science and engineering from the Institute of Metal Research, Chinese Academy of Sciences in 2008. His expertise is in sustainable functional materials, thermoelectrics, advanced manufacturing, and advanced microscopy. He is a highly cited Clarivate researcher with 450 peer-reviewed publications, which have attracted a Scopus citation of >34,500 and an H index of 100.

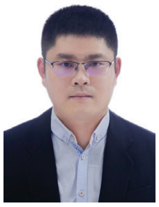

**Jianxun Ding**, *Changchun Institute of Applied Chemistry, CAS, China*

Dr. Jianxun Ding received his Ph.D. in Polymer Chemistry and Physics from Changchun Institute of Applied Chemistry, Chinese Academy of Sciences, P. R. China, in 2013. From 2017 to 2019, He worked as a Postdoctoral Research Fellow at Brigham and Women's Hospital, Harvard Medical School. His research focuses on synthesizing biodegradable bioactive polymers, developing bioresponsive polymer platforms for controlled drug delivery, exploiting polymer-based adjuvants for immunotherapy, and preparing polymer scaffolds for regenerative medicine. He has published over 150 scientific publications and authorized over 30 patents.

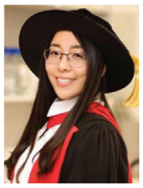

**Jing Jing Duan,** *Nanjing University of Science and Technology, Nanjing, China*

Prof. Jing Jing Duan achieved her Ph.D. from School of Chemical Engineering and Advanced Materials, Center for Materials in Energy and Catalysis, at the University of Adelaide in 2016. During 2016 to 2019, Dr. Duan worked as a Research Fellow in Prof. Chuan Zhao's group at the University of New South Wales, Prof. Allen J. Bard's group (Father of Modern Electrochemistry) at the University of Texas at Austin, Prof. Shi Zhang Qiao's group at the University of Adelaide. Then she joined School of Energy and Power Engineering at Nanjing University of Science and Technology as a full professor since 2020. Prof. Duan's research interests focus on developing clean energy technologies to neutralize carbon emission, including fuel cells, water/$N_2$/$CO_2$ electrolysis *et al*. She is also devoted to investigating reaction mechanisms by a combination of operando techniques and multiscale simulation. Prof. Duan has received several awards, for example, the 2021 JMCA Emerging Investigator, 2021 National Young Talents, 2020 Top 2% Scientists Globally, 2018 Women's Research Excellence, 2017 Postgraduate Alumni Medal of the University of Adelaide, *etc*.

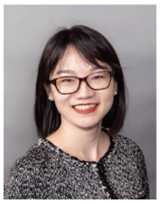

**Zi (Sophia) Gu**, *University of New South Wales, Sydney, Australia*

Zi (Sophia) Gu is an Associate Professor at the School of Chemical Engineering, University of New South Wales (UNSW Sydney). She leads a NanoBiotechnology Research Group at UNSW, and is currently a principal investigator in Australian Centre for NanoMedicine and UNSW RNA Institute. After obtaining PhD at the University of Queensland, she secured a NHMRC Fellowship and joined UNSW in 2016. Her research focuses on developing advanced nanomaterials and delivery strategies to address critical issues in health and medicine. To date, she has published over 80 journal papers in the areas of nanomaterials and nanomedicine.
Her research focuses on developing advanced nanomaterials to address critical issues in health and medicine. She utilises interdisciplinary approaches that integrate material chemistry, biomedical engineering and nanotechnology to synthesise functional nanoparticles, optimise their biomedical properties, and develop advanced delivery nanoparticles, smart bioimaging probe, and other bio-functional nanomaterials. She has published over 70 research papers on *Advanced Materials*, *Advanced Functional Materials*, *Advanced Science*, *Small*, *Small Methods*, *Biomaterials* etc., with a total citation of >3500 and H-index of 34 (Google scholar, Mar 2023). She has been awarded competitive grants from ARC, NHMRC and NSW Health (>1M as Lead CI in recent 3 years). She organised several international nanomedicine and advanced materials conferences. She serves as an Associate Editor for *Exploration* and *Journal of Nanobiotechnology*, and an active reviewer for ARC and NHMRC.

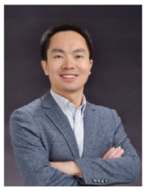

**Yuanyu Huang**, *Beijing Institute of Technology, Beijing, China*

Dr. Yuanyu Huang received his Ph.D. degree in Biotechnology from Peking University in 2013. After 3 years postdoctoral training, he joined Beijing Institute of Technology in 2016 as an Associate Professor and Principal Investigator. He was prompted to a Professor in 2020. Dr. Huang's primary research interest focuses on the development of nucleic acid-based therapeutics, including designing and optimizing nucleic acid molecules (siRNA, mRNA, Aptamer, CRISPR/Cas, etc.), exploring innovative drug delivery system and their translational medicine applications in the treatment of diverse diseases. He has published more than 70 scientific publications and filed more than 10 patents, including two authorized PCT applications.

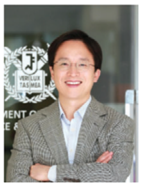

**Ho Won Jang**, *Seoul National University, Seoul, Korea*

Ho Won Jang is a full professor in Department of Materials Science and Engineering at Seoul National University. He earned his Ph.D. from the Department of Materials Science and Engineering of Pohang University of Science and Technology in 2004. He worked as a research associate at University of Madison-Wisconsin from 2006 to 2009. Before he joined Seoul National University in 2012, he worked at Korea Institute of Science and Technology as a senior research scientist. He is a member of Young Korean Academy of Science and Technology. His research interests include materials synthesis and device fabrication for solar fuel generation, chemical sensing, nonvolatile data storage, neuromorphic computing, plasmonics, ferroelectronics, and metal-insulator transition. He has published more than 430 papers in international refereed journals.

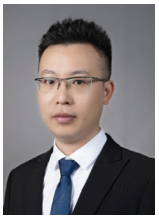

**Xiaoyuan Ji**, *Tianjin University, Tianjin, China*

Prof. Xiaoyuan Ji is the full professor in Tianjin University and the deputy director of the Engineering Research Center of the Ministry of Education for Intelligent Medical Engineering. He received his PhD degree at Institute of process engineering, Chinese Academy of Sciences and Harvard University. His research interest is focused on biomaterials, nanomedicine and nanobiotechnology, which involve the design, fabrication, biomedical application and clinical translation of versatile biomaterials, including heterojunction nanomedicine, micron/nano robot, drug/gene delivery, nanodynamic therapy, nanocatalytic therapy, and localized disease therapy. He has published many papers in top journals including Nature Communications, Science Advances, Advanced Materials, Angewandte Chemie International Edition et al. Prof. Ji has received multiple awards/grants including the Outstanding Youth Foundation (National Natural Science Foundation), Chinese Medical Science and Technology Award, Clarivate's Global Highly Cited Researchers List (2022), World's Top 2% Scientists (from Mendeley Data, Elsevier; 2021, 2022). He also serves on numerous editorial boards including as Associate Editor of *Exploration*, Editorial Board Member of *The Innovation*, *Chinese Chemical Letters*, *Current Medicinal Chemistry* et al.

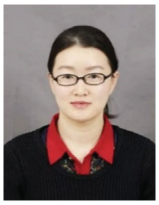

**Linlin Li**, *Beijing Institute of Nanoenergy and Nanosystems, Chinese Academy of Sciences, Beijing, China*

Linlin Li received her Ph.D. degree from the Technical Institute of Physics and Chemistry, Chinese Academy of Sciences in 2008. Now She is a full professor and PI in Beijing

Institute of Nanoenergy and Nanosystems, Chinese Academy of Sciences. Her research interest focuses on the smart biomaterials, nanomedicines and self-powered bioelectronics for disease therapy, regenerative medicine, and biosensing. She has published over 140 peer-reviewed journal articles. These publications have attracted more than 11,000 citations (h-index of 48). She now serves as Associate Editor of *Exploration* and *Chinese J. Anal. Chem.*, Academic Editor of The Innovation, and Editorial Board Member of *BMEMat*.

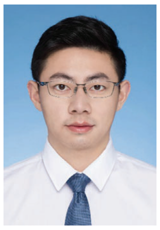

**Han Lin**, *Shanghai Institute of Ceramics Chinese Academy of Sciences, Shanghai, China*

Han Lin received his Ph.D. degree from Shanghai Institute of Ceramics Chinese Academy of Sciences (SICCAS), in 2019. He is now an associate professor at SICCAS, and a Principal Investigator at Shanghai Tenth People's Hospital of Tongji University. His research interest is in the design and synthesis of low-dimensional biomaterials, revealing nano-bio interactions (theoretical modeling and mechanism studies), and exploring their various biomedical applications including cancer theranostics, infectious disease, bone regeneration and so on. He has authored and co-authored over 70 peer-reviewed academic papers in highly reputed journals. Google Scholar gives a citation of 9,000 times with a h-index of 41.

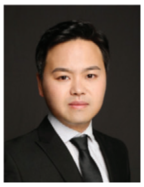

**Daishun Ling**, *Shanghai Jiao Tong University, Shanghai, China*

Prof. Daishun Ling received his Ph.D. in School of Chemical and Biological Engineering, Seoul National University. Later, he worked as a professor in the College of Pharmaceutical Sciences, Zhejiang University. Now, he is a full professor in School of Chemistry and Chemical Engineering, Shanghai Jiao Tong University. Prof. Ling's primary research interest has been focusing on the assembly/disassembly chemistry, stimuli-responsive biomaterials, dynamic nanoassembly based drug delivery systems. Up to now, he has published > 100 papers in prominent international journals including *Nature Nanotechnol., Nature Mater., Nature Biomed. Eng., Nature Commun., J. Am. Chem. Soc., Angew. Chem. Int. Ed., Adv. Mater., Adv. Sci., Adv. Funct. Mater., Nano Today, ACS Nano, Nano Lett., ACS Cent. Sci.*, etc. He has been serving as the Associate Editor of *Exploration* and *Nano Select*, Assistant Editor of *J. Control Release*, Theme Editor of *Adv. Drug Deliv. Rev.*, Academic Editor of *The Innovation*, and the Editorial Board Member of *Sci. Bull*.

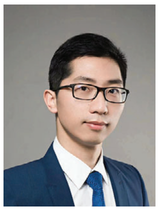

**Chengliang Wang**, *Huazhong University of Science and Technology, Wuhan, China*

Chengliang Wang is a full professor at the Huazhong University of Science and Technology. He received his bachelor's degree from the Nanjing University in 2005 and Ph.D. degree from the Institute of Chemistry, Chinese Academy of Sciences, in 2010. He then worked at the Chinese University of Hong Kong, the University of Muenster, and the Technical University of Ilmenau till 2016. He then joined in the Huazhong University of Science and Technology as a full professor. He is working on novel conjugated organic and polymeric materials for optoelectronics and batteries and particularly focuses on green rechargeable batteries, sustainability, recyclability and carbon neutralization.

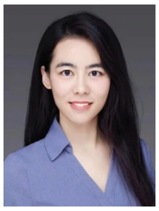

**Jiajia Xue**, *Beijing University of Chemical Technology, Beijing, China*

Jiajia Xue received her Ph.D. degree from Beijing University of Chemical Technology (under the supervision of Prof. Liqun ZHANG) in 2015 and conducted her postdoctoral research in the Department of Biomedical Engineering at Georgia Institute of Technology from 2015 to 2019 (co-advisor: Prof. Younan XIA). She is now a professor at Beijing University of Chemical Technology. Her main research interests include the design of electrospun fiber scaffolds, hydrogels, and elastomer materials for tissue repair and replacement. She has published over 70 peer-reviewed papers, with a citation over 6,000 times. She is the Emerging Investigator in *Journal of Materials Chemistry B* of Royal Society of Chemistry in 2022. She has also received the 2023 Chinese Association of Biomaterials (CAB) Young Investigator Award.
